# Gut microbiota derived indoles are altered and associate with immune activation in moderate and severe carotid stenosis

**DOI:** 10.3389/fimmu.2026.1796312

**Published:** 2026-07-16

**Authors:** Kristine Stø, Karolina Skagen, Vigdis Bjerkeli, Per Magne Ueland, Johannes R. Hov, Marius Trøseid, Pål Aukrust, Thor Ueland, Bente Halvorsen, Mona Skjelland

**Affiliations:** 1 Institute of Clinical Medicine, Faculty of Medicine, University of Oslo, Oslo, Norway; 2 Department of Neurology, Oslo University Hospital, Oslo, Norway; 3 Research Institute of Internal Medicine, Oslo University Hospital, Oslo, Norway; 4 Department of Clinical Science, University of Bergen, Bergen, Norway; 5 Norwegian PSC Research Center and Section of Gastroenterology, Department of Transplantation Medicine, Oslo University Hospital, Oslo, Norway; 6 Section of Clinical Immunology and Infectious Diseases, Department of Rheumatology, Dermatology and Infectious Diseases, Oslo University Hospital, Oslo, Norway; 7 Section Clinical Immunology and Infectious Diseases Oslo University Hospital, Oslo, Norway

**Keywords:** carotid atherosclerosis, indoles, ischemic stroke, microbiome & dysbiosis, TMAO (trimethylamine N-oxide)

## Abstract

**Background:**

An imbalance of gut microbiota, their metabolites as well as inflammatory mediators have been increasingly linked to both atherosclerosis and stroke. However, data on microbiota derived tryptophan and histidine metabolites in carotid atherosclerosis are scarce.

**Purpose:**

We investigated serum microbiota derived indoles, imidazole propionate (ImP) and trimethylamine N-oxide (TMAO), representing three distinct gut bacterial-related metabolites, in patients with carotid atherosclerosis compared with healthy controls with normal findings on carotid ultrasound. We aimed to examine their relation to plaque characteristics, immune activation markers and traditional cardiovascular risk factors.

**Methods:**

Thirty patients scheduled for carotid endarterectomy and 18 control subjects were included in this cross-sectional study. Carotid arteries were investigated with ultrasound. Indoles, ImP and TMAO were analyzed by liquid chromatography-tandem mass spectrometry, and Lipopolysaccharide (LPS) by a Limulus Amebocyte Lysate chromogenic assay.

**Results:**

Compared to controls, patients exhibited lower levels of indole-3-propionic acid (IPA) (p=0.004) and indole-3-acetic acid (IAA) (*p* = 0.030). In patients, higher levels of indole metabolites were associated with lower C-reactive protein. ImP and TMAO did not differ between patients and controls.

**Conclusion:**

Patients with carotid atherosclerosis exhibited reduced serum concentrations of IPA and IAA, thought to have anti-inflammatory effects, and increased inflammatory markers, possibly suggesting disruptions in the gut–vascular-immune axis.

## 1 Introduction

Atherosclerosis is a chronic inflammatory disease characterized by accumulation of lipids and immune cells, and extracellular matrix remodeling (1, 2). Carotid atherosclerosis is a major cause of ischemic stroke due to plaque rupture and embolization, with carotid endarterectomy (CEA) as standard surgical treatment for symptomatic high-degree stenosis (3). However, the pathogenic mechanisms of carotid atherosclerosis and its transition to an unstable lesion is still incompletely understood, hampering the development of novel biomarkers for risk prediction and novel targets for therapy and prevention.

Emerging evidence indicates that a dysregulated gut microbiota influences host metabolic and cardiovascular risk through the production of metabolites which reach the systemic circulation and the vascular lesion (4–7). These metabolites can promote atherosclerosis through various mechanisms such as modulation of lipid metabolism, endothelial function and inflammation. Among the most well reported microbiota derived metabolites associated with atherosclerotic burden and especially coronary artery disease is trimethylamine N-oxide (TMAO), generated from dietary choline and carnitine and oxidation of TMA in the liver (4–7).

Indoles are metabolites generated from dietary tryptophan by gut bacteria and exert effects on inflammation and atherosclerosis-related processes and are in general considered beneficial (8). Indole-3-proprionic acid (IPA) seems to have antioxidant and anti-inflammatory properties and to reduce atherosclerotic plaque formation and indole-3-Aldehyde (IAld) reduces macrophage activation and atherosclerotic plaque inflammation (9). Indole-3-acetic acid (IAA) has been shown to restore the imbalance between the inflammatory M1 and pro-resolving M2 macrophages resulting in an anti-inflammatory net effect (10). On the other hand, Indoxyl sulfate (IS3) is regarded as a uremic toxin, and to have pro-inflammatory effects, promote endothelial dysfunction and enhance macrophage cell formation (8).

In addition, imidazole propionate (ImP), a metabolite produced from dietary histidine by gut bacteria, has been highlighted in recent studies (11–13) and shown to impair insulin signaling and to promote vascular inflammation, macrophage activation and atherosclerotic plaque formation. Elevated circulating ImP levels have been observed in patients with diabetes and to correlate with cardiovascular risk factors (11). In general, however, data on circulating microbiota derived tryptophan and histidine metabolites in carotid atherosclerosis are scarce or even lacking.

The aim of the present study was to provide new insights into the gut-immune axis in carotid atherosclerosis by examining circulating levels of indoles, ImP and TMAO in 30 patients undergoing CEA compared with 18 healthy controls with normal findings on carotid ultrasound, with a particular focus on indoles that potentially possess anti-inflammatory properties (8–10).

## 2 Materials and methods

### 2.1 Patients and control subjects

The study population has been described in a previous publication (14). In brief, this cross-sectional observational study was conducted between August 2017 and June 2019. Thirty patients with severe carotid atherosclerosis, defined as more than 50% stenosis, who were scheduled for CEA and 18 healthy control subjects, based on disease history, no regular use of any medication as well as no atherosclerosis on carotid ultrasound were included in this sub-study (**Table 1**). Patients were investigated on admission for CEA. The study was approved by the Norwegian Regional Committees for Medical and Health Research Ethics (ID REC 2017/2202 A) and was performed in accordance with the Declaration of Helsinki. All the participants gave written informed consent before inclusion.

**Table 1:** Baseline characteristics of patients and healthy controls.

	Controls N=18	Patients N=30	*P*-value
Age, (years) *	60.9 (6.1)	70.1 (9.8)	0.001
Male sex	27.8 (5)	43.3 (13)	0.28
Body Mass Index (kg/m^2^) *	24.9 (3.1)	26.4 (4.4)	0.221
Waist-hip ratio (cm/cm)	0.89 (0.076)	0.98 (0.076)	0.002
Hypertension	0 (0)	70 (21)	<0.001
Statin treatment	0 (0)	76.7 (23)	<0.001
Platelet inhibitor	0 (0)	90.0 (27)	<0.001
C-reactive protein, (mg/L)*	0.9 (0.6)	4.9 (4.9)	*<0.001*
Leukocyte count, (10^9^/L)*	4.9 (1.1)	9.5 (2.2)	*<0.001*
Total cholesterol, (mM)*	5.4 (0.64)	3.7 (0.81)	<0.001
LDL cholesterol, (mM)*	3.4 (0.63)	2.1 (0.75)	<0.001
HbA1c (%)*	5.2 (0.28)	5.8 (0.88)	*0.017*
Antibiotics last 3 months	0 (0)	20.0 (6)	0.067
History of heart disease	0 (0)	30 (9)	0.007
Current smoker	5.6 (1)	16.7 (5)	0.23
Degree of stenosis > 70%	-	73.3 (22)	-
Heterogenous or echolucent plaque	-	63.3 (19)	-
MRI DWI** lesion	-	70.0 (21)	-
CEA within 14 days	-	72.0 (18/25)	-
CEA within 7 days	-	60 (15/25)	-

Values are given as % (n) or *mean (SD). **MRI DWI magnetic resonance imaging diffusion-weighted imaging.

### 2.2 Clinical characteristics, blood sampling and carotid ultrasound

As previously described (14), information on general health and diet was collected from questionnaires and medical journals. Anthropometric measurements, serum sampling (fasting state), and bilateral carotid ultrasound were performed at inclusion. Plaques were categorized as predominately calcified, heterogenous or echolucent and, the severity of stenosis as either 50-69% or >70%.

### 2.3 Analyses of tryptophan-derived indole metabolites, ImP and TMAO

Circulating metabolite quantification of the microbiota derived indoles, ImP and TMAO was conducted by Bevital, Bergen, Norway, using chromatography-tandem mass spectrometry (LC-MS/MS). Serum samples were processed immediately and stored at −80 °C until analysis. Stable isotope-labeled internal standards were used for accurate absolute quantification, and calibration curves with quality controls were included in each batch to monitor precision, accuracy, and stability (15, 16).

### 2.4 Lipopolysaccharide

Endotoxin, LPS, was quantitated with Limulus amebocyte lysate (LAL) assay using Pierce Chromogenic Endotoxin Quant kit (A395529) according to the manufacturer’s instructions, with the following modifications: Serum was diluted ten-folds and preheated to 70 °C for 10 min to inactivate inhibitors in the assay. Endotoxin-free tubes and water were used in the assay.

### 2.5 Plaques

Carotid plaques were removed *en* bloc during surgery and stored immediately in Allprotect Tissue Reagent (Qiagen, Hilden, Germany) at room temperature for up to 14 days before being frozen at -80 °C until further analysis. Each frozen plaque was placed in a sterile dish and then held by a sterile forceps allowing the tissue reagent to drain off before being transferred to a new sterile dish where residual reagent was removed, before being weighed.

### 2.6 Statistics

Descriptive statistics are given as number and proportion (%), mean with standard deviations or median (min-max). For demographic variables (**Table 1**), independent sample-t-test was used to compare continuous variables while chi-square or Fisher’s exact test were used to compare proportions. Spearman correlation was used to evaluate relationships between variables. Endotoxin, neopterin, IPA, IAA, IS3 and ImP were skewed and log10 transformed prior to analysis. As most variables were associated with age and age was different between the groups, we used multivariate regression with age as a covariate to compare differences between patients and controls. In addition, statin and platelet inhibitor use were also included as covariates. P-values for this analysis were Bonferroni adjusted for the 10 assessed metabolites. Firth logistic regression was used to evaluate if assessed metabolites were independently associated with CEA in a model including metabolites, CRP and age. *P*-values are two-sided and considered significant when <0.05. IBM SPSS Statistics for Windows, statistical software version 25.0 (IBM Corp., Armonk, NY, USA) was used for data analyses.

## 3 Results

### 3.1 Baseline characteristics of patients and healthy control subjects

Demographic and clinical characteristics are presented in **Table 1**. Patients were older than healthy controls (*p* < 0.001), but with no differences in sex, current smokers and body mass index. As expected, the patients had more risk factors for cerebrovascular disease such as hypertension and diabetes and increased inflammatory markers, i.e., C-reactive protein (CRP) and leukocyte counts. Regarding medications, 76% of the patients used statins, 90% used platelet inhibitors and 20% had used antibiotics within the last three months prior to study inclusion, but none during the last three weeks. Of the 30 patients, 25 were symptomatic within the last 1.5 months (stroke, TIA or amaurosis fugax) and 21 had MRI scans showing acute infarction. One had a small infarction 5.5 months ago, whereas four had unspecific cerebrovascular symptoms. Sixty percent (15/25) underwent CEA within seven days for their ischemic event, and further three within 14 days, and the remaining between 14 and 45 days. Concerning findings on carotid ultrasound, 22 (73%) patients had high degree carotid stenoses (>70%), and for 19 (63%) of the patients, plaques were characterized as either heterogenous or echolucent, i.e., morphologic features associated with plaque instability (3).

### 3.2 Microbiota derived metabolites and endotoxin

**Figure 1:**
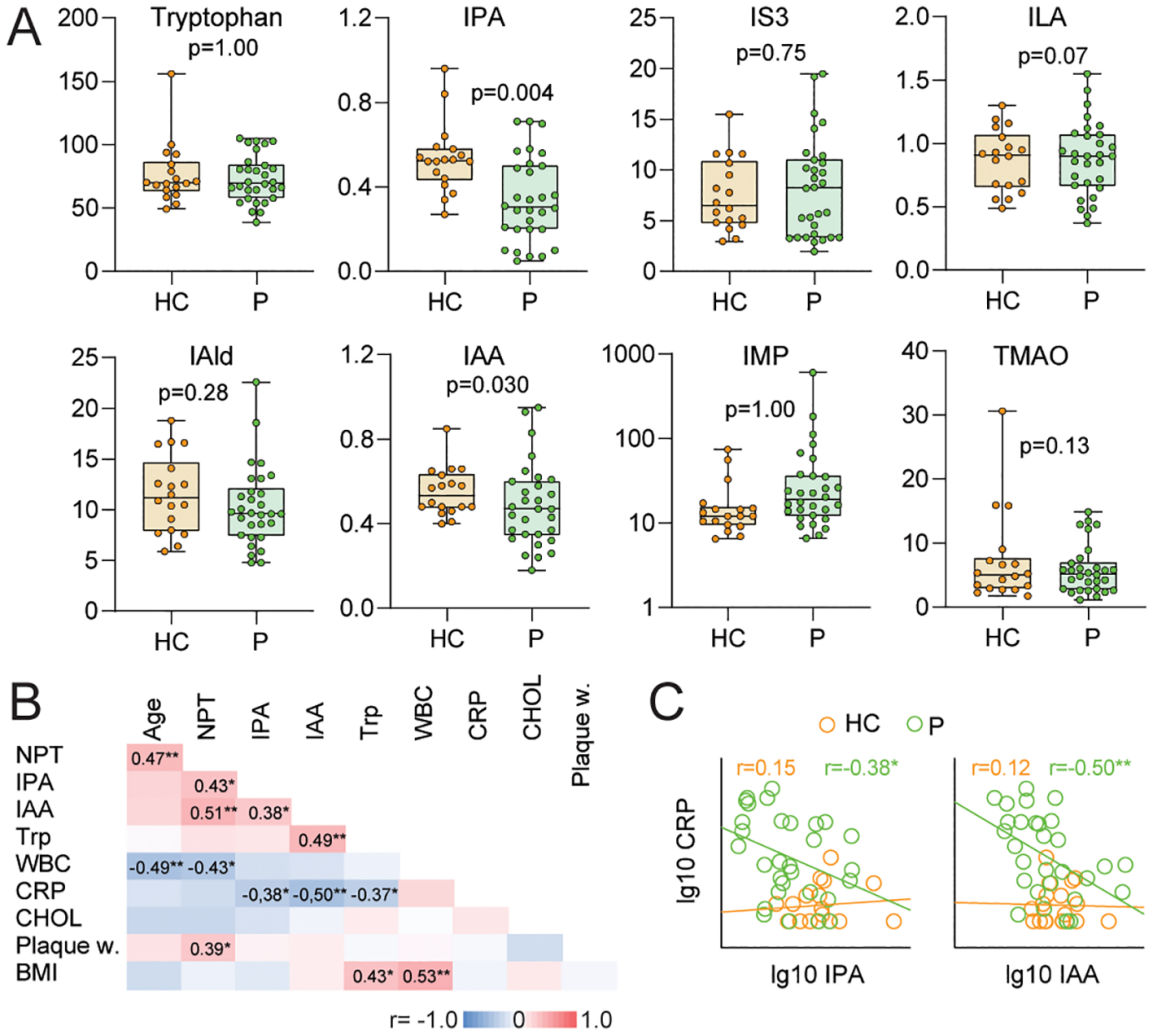
Microbiota metabolites in patients with carotid atherosclerosis (P) (green) and controls (HC) (yellow). **(A)** Box plots showing the difference between individual patients and controls for tryptophan (µmol/L), indole metabolites (µmol/L), ImP (nmol/L) and TMAO (µmol/L), *p*-values adjusted for age, platelet inhibitor use and statin use. **(B)** Associations between IPA and IAA with age, neopterin (NPT) Tryptophan (Trp), white blood cell count (WBC), CRP, cholesterol (CHOL) and BMI in the patient group, *p<0.05, **p<0.01. **(C)** Correlations of IPA and IAA with CRP in patients and controls.

Compared to controls, patients had significantly lower levels of IPA (*p* = 0.004) and IAA (p=0.030), adjusted for age, statins and platelet inhibitors. There were no differences for the remaining indoles (IS3, ILA and IAld), tryptophan, ImP or TMAO (**Figure 1A**).

Firth logistic regression was used to evaluate if assessed metabolites were independently associated with CEA in a model including metabolites, CRP and age. In this model, age/10 (OR = 7.54 [95% CI 2.12 - 65.57], *p* < 0.001), normalized log CRP (OR = 16.61 [95% CI 2.69 - 131.52], *p* < 0.001) and normalized log IPA (OR = 0.23 [95% CI 0.02 - 0.90], *p* = 0.031) were significant, but not normalized log IAA (OR = 1.09 [95% CI 0.31 - 4.10], *p* = 0.89). Thus, while age and systemic inflammation correlated with these indoles, at least IPA seems to show an independent association with CEA.

LPS was detectable (i.e. >detection limit) in one control subject (6%) and in 14 (47%) of patients (Chi square *p* < 0.01).

### 3.3 Correlations of the downregulated indole metabolites and markers of inflammation, LPS and plaque characteristic

In patients, IPA and IAA were negatively correlated to CRP (**Figures 1B, C**). This pattern was not seen in healthy controls. No correlations were found between these metabolites and LPS, neither when comparing the quantitative levels of the measured microbial metabolites between the subset of patients with detectable LPS and those with non-detectable LPS (**Supplementary Table 1**).

There were no correlations between the indole metabolites and recent symptoms, infarction on MRI, degree of stenosis, or plaque characteristics.

## 4 Discussion

In this cross-sectional study we investigated circulating microbiota derived metabolites of tryptophan (indoles), histidine (ImP) and carnitine/choline (TMAO) and markers of immune activation in patients with severe carotid atherosclerosis who underwent CEA and in healthy controls. Our main findings were that patients had lower levels of IPA and IAA, both indole metabolites of tryptophan.

IPA has been found to be downregulated in patients with atherosclerotic disorders (17) and interestingly, Li et al. found that higher plasma tryptophan and IPA levels were associated with lower risks of cardiovascular and all-cause mortality, supporting a beneficial effect of high IPA levels. Low IPA levels have been associated with carotid artery plaque in women with and without HIV infection (18), but in general data on IPA in relation to carotid atherosclerosis and stroke are lacking. In this present study we showed that patients with carotid atherosclerosis had lower serum levels of IPA compared with healthy controls. IPA levels were inversely correlated with CRP in the patient group, but not in the healthy controls, which may further reflect an anti-inflammatory effect of IPA (9). The potential beneficial effects of IPA in relation to atherosclerosis seems not be restricted to its anti-inflammatory properties, but also to be related to its ability to promote reverse cholesterol transport (19). A similar pattern was found for IAA, but not for the other indole metabolites, i.e., IS3, ILA and IAld.

Macrophages play a central role in atherosclerosis and can adopt pro- or anti-inflammatory phenotypes. M1 macrophages promote plaque inflammation and instability, whereas M2 macrophages support tissue repair and plaque stabilization (10). Notably, IPA and IAA, which were downregulated in patients, are both involved in M2 macrophage polarization in atherosclerosis and inhibit inflammation (10, 20), consistent with their negative correlation to CRP.

In this current study we found no significant difference between patients and controls for TMAO and ImP after adjustment for age, platelet inhibitors and statins. Although TMAO has been implicated in atherosclerosis and cardiovascular risk in several large studies (4–7), the literature is somewhat inconsistent and higher TMAO levels have also been associated with a lower risk of cardiovascular events (7). However, the lack of correlation might be influenced by diet, timing of sampling in relation to the clinical event and not least sample size of our study. ImP is a potential emerging metabolite which has been shown to promote atherogenesis (11) and to predict cardiovascular risk in patients with coronary artery disease (12). Nevertheless, data on ImP in patients with carotid atherosclerosis and stroke are very limited, thus further studies in larger cohorts are needed to clarify the role of ImP in these patients.

## 5 Limitations and strengths

This study benefits from a well-characterized cohort in which all participants, including control subjects, underwent both ultrasound and fasting blood tests, allowing for integrated imaging and biochemical analyses. The simultaneous measurement of microbiota derived metabolites and inflammatory markers strengthens the study’s ability to explore gut-vascular-immune interactions. Nevertheless, a major limitation is the relatively small sample size, raising concerns about type II errors for negative findings, e.g., TMAO. Additionally, the cross-sectional design precludes conclusions regarding causality and, correlations should be interpreted with caution. We also lack longitudinal data related to clinical outcomes. Finally, although the LAL assay remains the regulatory and diagnostic gold standard for broad-spectrum endotoxin detection, in particular as LPS molecules vary greatly depending on bacterial strains, we lack data from supplementary more specific methods. In LAL method, the time for preheating at 70°C could have been too short.

## 6 Conclusion

Altered gut microbiota derived tryptophan metabolites may be associated with immune activation and plaque characteristics in severe carotid atherosclerosis, possibly suggesting links to ischemic stroke beyond traditional cardiovascular risk factors.

## Data Availability

The raw data supporting the conclusions of this article will be made available by the authors, without undue reservation.

## Supplementary material

Supplementary Table 1 Analysis of the measured metabolites according to LPS dichotomy.
