# Supplementary material for: Gut microbiota derived indoles are altered and associate with immune activation in moderate and severe carotid stenosis
Source: Front Immunol. 2026 Jul 16;17:1796312. doi: 10.3389/fimmu.2026.1796312 (PMC13422166; doi:10.3389/fimmu.2026.1796312)
Supplement: Supplementary Table 1 — Analysis of the measured metabolites according to LPS dichotomy. [file DataSheet1.pdf]

Supplementary table 1: Analysis of the measured metabolites according to LPS dichotomy.

	EMM	Controls low 95% CI	high 95% CI	EMM	CEA patients low 95% CI	high 95% CI	P
Trp	71,1	61,9	81,8	70,3	60,5	81,7	0,9
IPA	2,31	1,83	2,91	2,06	1,60	2,64	0,496
IS3	9,07	6,67	11,46	7,31	4,74	9,87	0,318
ILA	0,86	0,71	1,02	0,90	0,74	1,06	0,752
Neopterin	25,9	21,4	31,3	25,4	20,7	31,0	0,887
IAld	10,28	8,21	12,35	10,33	8,11	12,55	0,974
IAA	3,10	2,45	3,93	3,03	2,36	3,90	0,894
IMP	28,5	17,5	46,3	20,6	12,2	34,6	0,36
TMAO	5,08	3,59	7,19	4,54	3,13	6,58	0,657
